# Thermal Image Restoration Based on LWIR Sensor Statistics

**DOI:** 10.3390/s21165443

**Published:** 2021-08-12

**Authors:** Jaeduk Han, Haegeun Lee, Moon Gi Kang

**Affiliations:** 1 Samsung Electronics, Suwon-si 16677, Gyeonggi-do, Korea; hjdplus@yonsei.ac.kr; 2 School of Electrical and Electronic Engineering, Yonsei University, Seoul 03722, Korea; leehaegeun@yonsei.ac.kr

**Keywords:** long wage infrared, image optimization, regularization, deconvolution, total variation

## Abstract

An imaging system has natural statistics that reflect its intrinsic characteristics. For example, the gradient histogram of a visible light image generally obeys a heavy-tailed distribution, and its restoration considers natural statistics. Thermal imaging cameras detect infrared radiation, and their signal processors are specialized according to the optical and sensor systems. Thermal images, also known as long wavelength infrared (LWIR) images, suffer from distinct degradations of LWIR sensors and residual nonuniformity (RNU). However, despite the existence of various studies on the statistics of thermal images, thermal image processing has seldom attempted to incorporate natural statistics. In this study, natural statistics of thermal imaging sensors are derived, and an optimization method for restoring thermal images is proposed. To verify our hypothesis about the thermal images, high-frequency components of thermal images from various datasets are analyzed with various measures (correlation coefficient, histogram intersection, chi-squared test, Bhattacharyya distance, and Kullback–Leibler divergence), and generalized properties are derived. Furthermore, cost functions accommodating the validated natural statistics are designed and minimized by a pixel-wise optimization method. The proposed algorithm has a specialized structure for thermal images and outperforms the conventional methods. Several image quality assessments are employed for quantitatively demonstrating the performance of the proposed method. Experiments with synthesized images and real-world images are conducted, and the results are quantified by reference image assessments (peak signal-to-noise ratio and structural similarity index measure) and no-reference image assessments (Roughness (Ro) and Effective Roughness (ERo) indices).

## 1. Introduction

Thermal imaging is being extensively used owing to the ongoing global pandemic of coronavirus disease 2019 (COVID-19). Fever is one of the prominent symptoms of COVID-19, and people infected by the virus are detected efficiently by LWIR thermal cameras (also known as microbolometer cameras). Infrared (IR) thermography converts radiation in the LWIR bands to thermal images (commonly referred to as thermograms), and allows for the detection of temperature variations. Visualization of thermal information benefits the recognition of the surroundings in some cases (e.g., low-light conditions or dark areas) because thermal imaging tends to provide more meaningful information in urban environments with huge thermal variations [1]. Therefore, LWIR imagers have been extensively used in medical, military, and security applications, As well as commercial applications, such as remote sensing [2,3], medical imaging [4], advanced driver-assistance systems [5], and face recognition [6,7,8].

Continuing advances in IR imaging have resulted in inexpensive, portable IR cameras, shutterless operations, and both cooled and uncooled, which can provide high-resolution thermograms [9]. Despite the advances and increasing demand for thermograms, thermal imagers still suffer from certain problems when measuring the thermal distribution within the field of view. Microbolometers are sensitive to the radiation emitted by objects in the scene, and the background radiation reflected by the objects in the scene, and the background radiation reflected by these objects. The relationship between the irradiation collected at the sensor and the temperatures of the imaged materials is nontrivial. For example, the material and surface properties of an object alter its emissivity. Similarly, the reflective properties of an object will vary the amount of background radiation reflected by the object and subsequently collected by the thermal imaging sensor. This variability can lead to errors in the measurement of an object’s temperature.

Additionally, the LWIR imaging sensor suffers from image degradation caused by its intrinsic limitations. First, a typical LWIR camera has a problem of severe noise in the framework without cooling systems. In general, LWIR cameras with cooling systems provide high-quality thermograms; however, applications that use handheld devices require minimized LWIR cameras [9]. Most LWIR imagers employ uncooled systems because of their size, weight, power, and price. A thermal camera without the cooler is usually referred to as a microbolometer. Second, residual non-uniformity (RNU, also known as fixed pattern noise (FPN)) is another degradation of the LWIR imaging sensor without a mechanical shutter. Typical LWIR cameras employ a shutterless imaging system that allows sensors to operate continually without the need for the mechanical shutter for NUC. The shutterless operation prevents external interruption yielding recalibration, and the changing thermal distributions can be treated based on the temperature measurements of the camera [10]. However, shutterless operations refer to the absence of a reference LWIR image typically used to cancel the non-uniformity, and RNU are observed owing to the low performance of the NUC [11].

Various conventional methods have been proposed to reduce the distortions of thermal imaging sensor [12,13,14]. These traditional methods considered adjacent pixels or referred data sequences to reduce the RNU. However, the characteristics or statistics of thermal images have seldom been employed to regulate thermal image processors, even if the statistics of LWIR images have been studied for a decade. Morris et al. [1] found that the power spectrum of a thermal image is predicted by the generalized Laplace distribution and suggested ranges of the parameter of the Laplace distribution. Goodall et al. [15] studied distortions unique to LWIR images, and found statistical models for measuring the degree of presetting non-uniformity in thermal images. Moreno-Villamarín et al. [16] analyzed common image distortions, such as blur, white noise, JPEG compression, and non-uniformity, in fused LWIR and visible light images, and proposed an opinion-aware fused image quality analyzer. Pezoa and Medina [11] proposed a model for RNU (or non-uniformity) present in IR focal plane arrays (FPAs) in the frequency domain (note that the RNU in the thermal imaging sensor is usually observed as a grid-like pattern and is different from those of complementary metal-oxide semiconductor (CMOS) sensors). In particular, the statistical regularities of LWIR [16] or fused visible-LWIR images [15] were derived based on natural scene statistics (NSS) of the visible light images, but the characteristics were not incorporated as prior information to solve optimization problems of thermal imaging.

**Figure 1:**
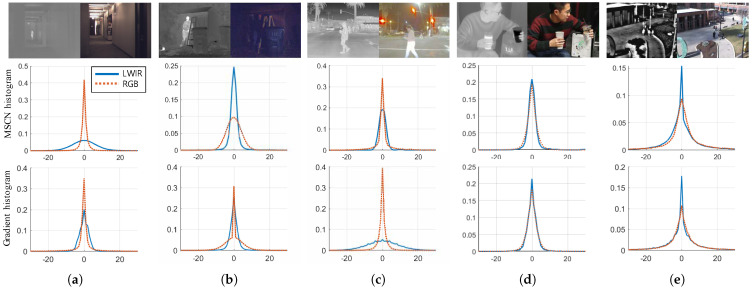
Test images of the five LWIR databases. The first row demonstrates different spectral images, thermal image, and visible light image, respectively. The second and third rows demonstrate the histogram and the histogram of the gradient distribution, respectively. Despite of their comparable field of view, the MSCN and the gradient distributions of LWIR and visible light images have been observed differently in most cases. (**a**) MORRIS [1]; (**b**) KRISTO [19]; (**c**) FLIR ADAS [5]; (**d**) SRIP; (**e**) OSU [20,21].

The thermal image processing algorithm can consider the prior knowledge, which is about the properties of LWIR images. Usually, the image restoration algorithm that considers visible light images attempts to incorporate the characteristics of the images, and the natural image statistics are usually derived from the distribution of gradients. For example, a mixture of the Gaussian approximation [17] and hyper-Laplacian [18] priors is derived from the heavy-tailed distribution of gradients in natural scenes and is incorporated as a regularization strategy. Although the relationship between the statistics and qualities of visible light images has been extensively studied, limited work has been conducted on the restoration of thermal images based on considerations of their own statistical characteristics. Our observation of LWIR images indicates that restoration of an LWIR image requires the inherent characteristics of the image. The spectral band of LWIR is characterized by wavelengths in the range of 8 to 14 μm and possesses its own statistics in the FPA. The difference between the gradients of visible light and LWIR images is shown in Figure 1 (note that we analyzed five datasets marked as MORRIS [1], KRISTO [19], ADAS [5], OSU1 [20], and OSU2 [21]). LWIR and visible light images have different mean-subtracted contrast normalized (MSCN) coefficients and gradient statistics even if they share a comparable field of view.

The experiments reported herein, present the properties of LWIR images without considering the LWIR imager’s optical or electronical characteristics. Therefore, we have tried to derive an intrinsic property using various datasets. A brief description of datasets follows. Dataset Morris [1] contains about 450 scenes with 350 outdoor and 100 indoor scenes. Each scene consists of a pair of images (visible light and LWIR image). Dataset Kristo [19] contains 180 scenes with 80 outdoor and 100 indoor scenes. Each scene also consists of a pair of images (visible light and LWIR image). Dataset ADAS [5] contains 14,452 annotated thermal images with 10,228 images sampled from short videos and 4224 images from a continuous 144-s video. All videos were taken on the streets and highways in Santa Barbara, California, USA from November to May. Dataset OSU [20] contains about 500 scenes obtained by surveillance cameras and each scene consists of a single LWIR image. Dataset OSU2 [21] contains about 10,000 scenes obtained by surveillance cameras and each scene consists of a pair of visible light and LWIR images. Finally, dataset SRIP contains 20 scenes, and each scene consists of a pair of visible light and LWIR images.

This paper is organized as follows: in Section 2, we derive a property of LWIR images using the aforementioned datasets. In Section 3, we propose an optimization strategy that incorporates our hypothetical property. In Section 4, the results of our optimization method are compared with those of the conventional methods. Finally, Section 5 concludes this study.

## 2. Related Work

### 2.1. Background

An underlying latent image is usually estimated under the assumption that the degradation is linear, and the degraded image can be restored by convex optimization. The degradation model of a thermal imaging camera is explained by a linear system, and the procedure is mathematically represented by a linear equation:

(1) y=Hx+n,

where y is an observed LWIR image, H is the system matrix of degradation, x is the clear latent image, and n is the corresponding error. When considering noise, the system matrix is considered to be an identity matrix I. In general, optical problems due to defocus aberration or atmospheric turbulence can be solved by an inverse operation of the system matrix but directly solving Equation (1) usually yields an unacceptable result.

The unacceptable result of the inverse matrix is caused by uncertainties. Therefore, the ill-posed problem is solved by using an iterative method. Many applications, such as medical imaging, surveillance, astronomy, and remote sensing, have adopted the iterative method based on convex optimization to reduce the uncertainty. Typically, a convex optimization problem to restore the degraded image is achieved by minimizing the following cost function as bellow:

(2) $$F(x)={\left|\left|y-\mathrm{Hx}\right|\right|}_{2}^{2}+\Gamma \left(x\right),$$

where the two terms indicate the data fidelity and regularization term, respectively. to constrain the feasible solution set, various regularization schemes have been deployed by the regularization term [22]. Therefore, in the case of optimization for the LWIR images, a regularization strategy related to the thermal images should be employed to obtain the steady-state solution.

### 2.2. Basic Concepts

**Figure 2:**
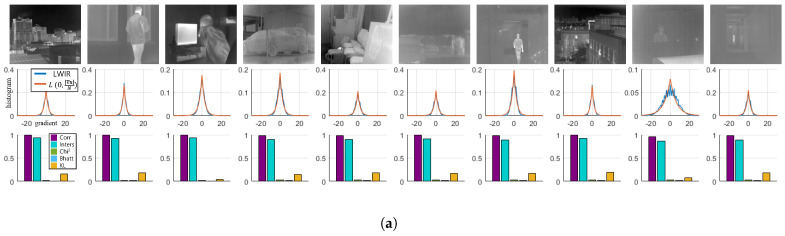

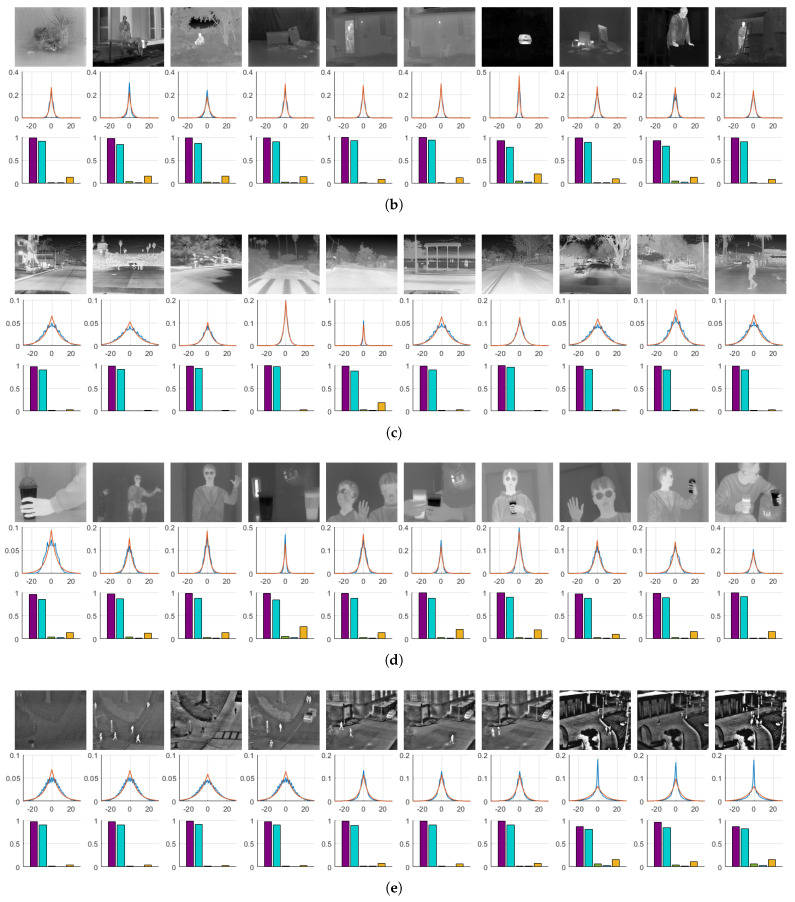
LWIR images, histograms, and the measured similarity. Ten images are sampled from each dataset, and the observed and estimated gradient distributions are demonstrated. The similarities between the observed and estimated distributions are also visualized. In most cases, measurement scores show that the two distributions are closely related. (**a**) MORRIS [1]; (**b**) KRISTO [19]; (**c**) FLIR ADAS [5]; (**d**) SRIP; (**e**) OSU [20,21].

An appropriately regularized cost function incorporates information regarding the restored images a priori defined as acceptable solutions and computationally stabilizes the solution of the under-constrained inverse problem [23]. As considerable work has been conducted to identify an efficient regularization strategy capable of the gradient distribution of the visible light images, an efficient image prior model, in which the intrinsic properties of LWIR images, needs to be constructed to recover the latent image. Therefore, we propose an optimization method incorporating the property of LWIR images derived from our observations on the datasets [1,5,19,20,21]. Our analysis of the LWIR images also departs from the gradient information. As in Figure 2, we estimate the gradient distribution of individual thermograms and attempt to demonstrate the correlation between the observed gradient distribution and the estimated distribution. All the histograms demonstrating the gradient distribution are visualized based on the kernels $\left[-1,1\right],{\left[-1,1\right]}^{T}$ yielding the output range [−510, 510] with 8-bit images.

Throughout our experiment for deriving natural statistics, the gradient distribution of individual LWIR images can be approximated by the following equation:

(3) $$h_{\nabla x}\left(n\right)∼\frac{N}{2\frac{\left|\nabla x\right|}{N}}\exp\left(-\frac{\left|n\right|}{\frac{\left|\nabla x\right|}{N}}\right),$$

where $h_{\nabla x}$ indicates a histogram demonstrating the gradient distribution of a thermogram x, and *N* denotes the number of pixels in the thermogram. Note that a random variable *Z* has a Laplace distribution if its probability density function (PDF) is as follows:

(4) $$Z∼\frac{1}{2b}\exp\left(-\frac{\left|z-\nu \right|}{b}\right):=Laplace\left(z|\nu ,b\right),$$

where ν is the mean, and b>0 is a scaling parameter. By using this abbreviation of the Laplace distribution, our observation can be hypothesized as below:

(5) $$\frac{h_{\nabla x}\left(n\right)}{N}∼Laplace\left(x_{n}|0,\frac{\left|\nabla x\right|}{N}\right),$$

which is a probability mass function of a zero mean Laplace distribution with a scaling parameter $b=\frac{\left|\nabla x\right|}{N}$. Additionally, to support our hypothesis formulated by Equation (5), five measures were utilized to show how the estimated distribution is different from the observed distribution, As summarized in Table 1. (For more detailed information about the measures, refer to [24,25].) The empirical scores for each measure are shown in Figure 2, and the average of their scores are presented in Table 2. As seen in Figure 2, the gradient distributions do not always satisfy Equation (5). Images with the severe pattern of RNU or saturation sometimes do not obey our hypothesis. For example, the dataset OSU often shows a saw pattern shape of the gradient distribution. Thermal images with saturated regions also record relatively low scores. Despite these perturbations, most of the thermograms can be considered as following our hypothesis.

The gradient distribution (or marginals) is assumed to be the Laplace distribution that allows image restoration algorithms to deploy total variation (TV) regularization [18]. The assumption of Laplace-distributed gradients in visible light images inspires a number of $L_{1}$-norm or TV-norm-based methods at the beginning [26], but recent work has focused on $L_{p}$-norm-based (0<p<1) methods capable of achieving heavy-tailed distribution [17,18] or nonconvex cost functions [27]. However, TV-regularization is a meaningful regularization strategy, and suitable for thermograms in accordance with our hypothesis of Equation (5). Therefore, the TV-regularized cost function for restoring LWIR images can be expressed as below:

(6) $$F(x)={\left|\left|y-\mathrm{Hx}\right|\right|}_{2}^{2}+\lambda \left|\nabla x\right|,$$

where λ is a regularization parameter, and ∇ denotes the first derivative operator.

Moreover, the Laplace distribution hypothesis of LWIR images enables additional studies. The regularization term in Equation (6) can incorporate additional prior knowledge derived from the statistical properties of the Laplace distribution. The mean and variance of the derivative signal ∇x are important clues that can be inferred from our hypothesis. First, the mean of ∇x is intuitively obtained owing to the symmetricity of the Laplace distribution:

(7) $$E\left[\nabla x\right]=\mu_{\nabla x}=0.$$

Second, the variance of ∇x is also obtained by referring to the properties of the Laplacian PDF as indicated below:

(8) $$Var\left[\nabla x\right]=2{\left(\frac{\left|\nabla x\right|}{N}\right)}^{2}.$$

Finally, another representation for the variance of the PDF whose mean is zero can be applied to our derivation based on Equation (7) as follows:

(9) $$Var\left[\nabla x\right]=\frac{||\nabla x-\vec{\mu_{\nabla x}}{\left|\right|}_{2}^{2}}{N}=\frac{{\left||\nabla x|\right|}_{2}^{2}}{N},$$

where $\vec{\mu_{\nabla x}}$ is a vector whose components are $\mu_{\nabla x}$, i.e., $\vec{0}$. The properties of Equations (7) and (9) originate from the symmetricity, and Equation (8) comes from the exponentiality of the Laplace-distributed gradient distribution.

The cost function that accommodates our hypothesis about the Laplace-distributed gradient histogram can be derived by integrating the properties of symmetry (or zero mean) and exponentiality. The properties obtained by integrating Equations (8) and (9) are as follows:

(10) $$\left|\nabla x\right|=\sqrt{\frac{N}{2}}{\left||\nabla x|\right|}_{2},$$

which means the relationship between anisotropic TV (|∇x|) and isotropic TV(${\left\|\nabla x\right\|}_{2}$) in thermal imaging. Finally, the regularization term in Equation (6) can be substituted by Equation (10), and the cost function suitable for the LWIR image is proposed as below:

(11) $$F(x)={\left|\left|y-\mathrm{Hx}\right|\right|}_{2}^{2}+\tilde{\lambda }{\left|\left|\nabla x\right|\right|}_{2},$$

where $\tilde{\lambda }$ is a re-scaled regularization parameter. Quantifying the isotropic functional like TV on grid $Z^{2}$ (which is not isotropic) is difficult, and anisotropic TV yields larger TV values owing to the metrication artifacts [28]. The LWIR image optimization takes advantage of isotropic TV which has more proper definition of discrete TV based on Equation (10).

In the next section, an advanced hypothesis based on patc-level analysis is derived by developing Equation (5), and a refined cost function incorporating patch-based gradient statistics is proposed. Finally, we propose an optimization method that minimizes the cost function by employing isotropic TV regularization.

## 3. Proposed Method

In this section, the gradient distributions are further expanded and applied to image optimization problems. The Laplace distribution can also describe not the gradient but the high-frequency contents of thermal images. We observe that the natural statistics of the thermal images are locally satisfied by performing a patch-level analysis. Henceforth, we propose an optimization method that employs observed patch-level natural statistics. The localized properties corresponding to Equations (7)–(9) are also derived and considered a regularization strategy. Note that the proposed optimization method inherits the framework of coordinate-wise optimization from our previous work [22] to accommodate local priors.

### 3.1. Natural Statistics of Thermal Images

**Figure 3:**
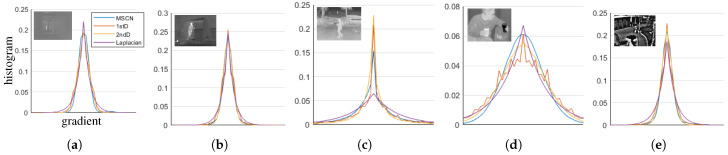
Distribution derived from the high-frequency of LWIR images. The statistics of the high-frequency is derived with three filters, MSCN, the first and the second derivatives. The three filtered signals demonstrate similar distributions. (**a**) MORRIS [1]; (**b**) KRISTO [19]; (**c**) FLIR ADAS [5]; (**d**) SRIP; (**e**) OSU [20,21].

In the restoration of visible images, the gradient statistics are mainly recognized as prior knowledge as in [17,29,30]. However, in the studies [15,16] of thermal images, researchers tried to explain the characteristics of thermal images by using the MSCN coefficients. Therefore, we also have conducted an experiment to estimate the histogram of features obtained using the MSCN coefficients. Figure 3 demonstrates the histogram estimation process of the MSCN coefficients in thermal images. Additionally, histograms of signals filtered with the first and second derivatives are also visualized with a log scale for a clearer comparison. The MSCN coefficients and the high frequencies filtered by the second derivative show similar distributions to the gradient distribution, and their histograms can also be estimated using the Laplace distribution. Table 3 demonstrates the mean of the scores obtained by the five measures from the 50 images in Figure 2. The scores of the three operators can be considered highly correlated with each other.

Finally, the natural statistics of thermal images can be derived from the results presented in Table 3. Our hypothesis indicates that the high-frequency components of the thermal image have a Laplace distribution. The first and second derivative operators, which can be utilized for the computation of image gradients, can be considered to filter out high frequencies. The MSCN coefficients can also be considered as an adaptive band-pass filter with a high-frequency band. Linear operators or adaptive filters that extract the details of the thermal image could be utilized to verify our hypothesis. For example, the image gradients filtered by the Sobel operator also obey the Laplace distribution.

### 3.2. Patch-Based Statistics

**Figure 4:**
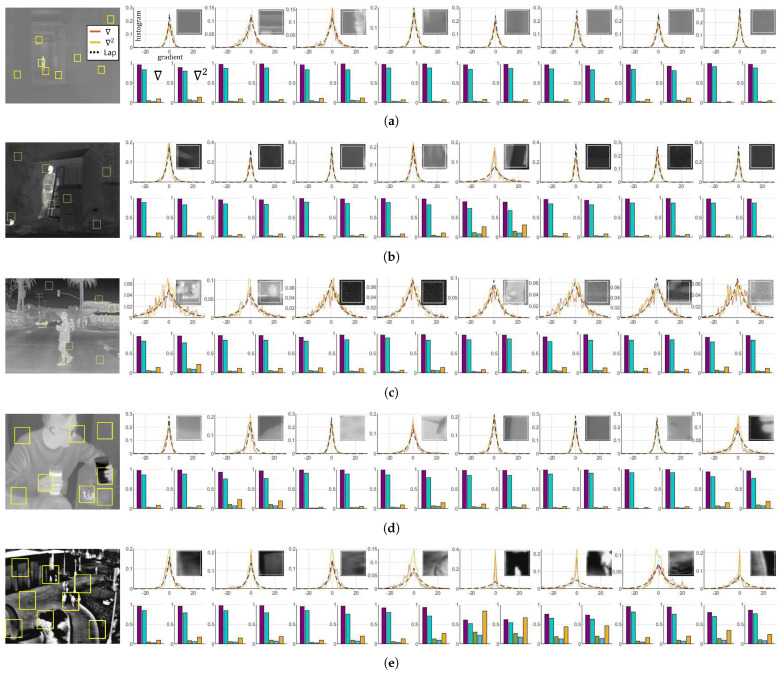
Analysis of the patch-based Gradient statistics. The patches (of size 21 × 21) are randomly sampled, and the observed and estimated distribution of high frequencies using ∇ and $\nabla^{2}$ have been analyzed with the measures of Table 1. All the LWIR images in the datasets have demonstrated similar results; most of the patches satisfy our hypothesis. (**a**) MORRIS [1]; (**b**) KRISTO [19]; (**c**) FLIR ADAS [5]; (**d**) SRIP; (**e**) OSU [20,21].

A further analysis is continued from the previous section in which the hypothesis is derived from the entire LWIR image. We have conducted an experiment based on patches that are randomly sampled from the LWIR images and observed that our hypothesis of Equation (5) is valid for image patches. A number of image patches were analyzed using the same approach as demonstrated in Figure 4; each gradient distribution was obtained by considering the information of individual patches. In addition to the first derivative, the second derivative (i.e., Laplacian operator) also used to derive the high-frequency components to verify our hypothesis. Therefore, Equation (5) can be rewritten by considering our patch-level experiment in Figure 4 as below:

(12) $$\frac{h_{\nabla^{t}P_{i}\left(x\right)}\left(n\right)}{l^{2}}∼Laplace\left(x_{n}|0,\frac{|\nabla^{t}P_{i}\left(x\right)|}{l^{2}}\right),$$

where t=1,2 used as a derivative index, $P_{i}\left(x\right)$ indicates a patch of an LWIR image x with a center pixel *i*, and l×l indicates the pixel size. As a result, following properties can also be derived from image patches. First, the properties of the symmetric distribution are As follows:

(13) $$<\vec{1},\nabla^{t}P_{i}\left(x\right)>=0,$$

and the property derived from the variance is written as follows:

(14) $$|\nabla^{t}P_{i}\left(x\right)|=\sqrt{\frac{N_{i}}{2}}\|\nabla^{t}P_{i}\left(x\right){\|}_{2},$$

where $N_{i}$ is the number of pixels in a single patch with a center pixel *i*. These properties are considered as local priors and incorporated into our optimization method as a regularization strategy.

**Figure 5:**
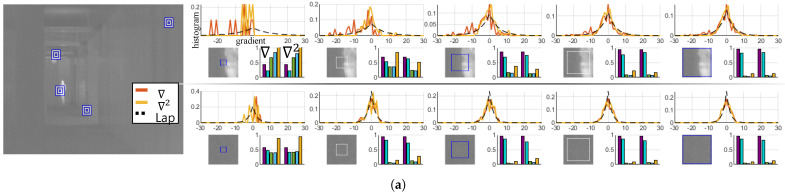

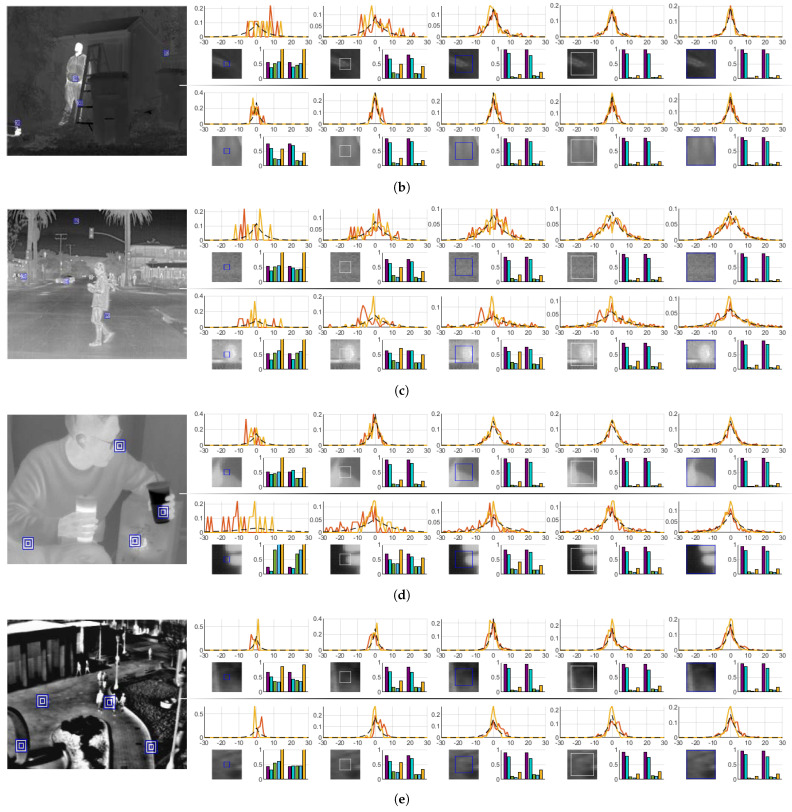
The validity of our hypothesis with varying patch sizes. The distribution of high-frequency components can be clearly estimated by the Laplace distribution as the size of the patch increases. Note that the measure scores over 1 were clipped for clear demonstration. (**a**) MORRIS [1]; (**b**) KRISTO [19]; (**c**) FLIR ADAS [5]; (**d**) SRIP; (**e**) OSU [20,21].

**Figure 6:**
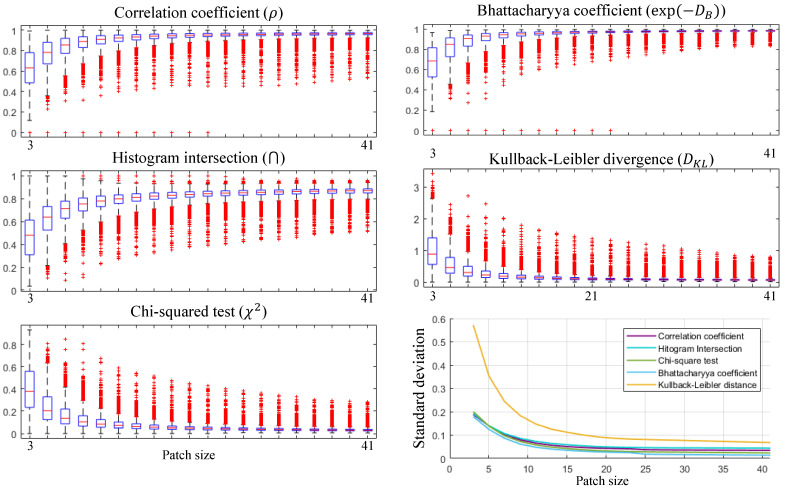
Experiment for deciding the optimal patch size. The interquartile range (IQR) and standard deviation were utilized to derive the appropriate condition for the properties of the LWIR image. A total of 4000 patches were randomly sampled from the datasets for each patch size. In this study, size of 21 × 21 has been empirically decided as the optimal patch size. IQRs of five measures with respect to increasing patch size are demonstrated. IQRs of each patch size were calculated based on the randomly sampled 4000 patches. Standard deviation with respect to increasing patch size is also demonstrated.

In our experiments, Equation (12) does not hold in all image patches as shown in Figure 4. Thus, an experiment has been conducted to investigate the validity of Equation (12). As seen in Figure 5, gradient distributions have been analyzed with respect to the patch size, and we have observed that the degree of the validity increases as the patch size increases. The scores of correlation coefficient, histogram intersection, and Bhattacharyya coefficient converge to the value of one, and the scores of the Chi-squared test and Kullback–Leibler divergence converge to zero as the number of pixels in a patch increases (note that the Bhattacharyya coefficient was utilized instead of the Bhattacharyya distance due to the absence of definition ln0). We have tried to deduce the optimal patch size analyzing the interquartile range (IQR) and standard deviation of the measures. With the patch size being varied, 4000 patches were randomly sampled from the datasets, and the IQR and standard deviation were calculated at each size as demonstrated in Figure 6. The blue boxes show the IQR (=Q3−Q1); the top line of the box shows the 75th percentile (Q3), the bottom line shows the 25th percentile (Q1), and the center line shows the 50th percentile, i.e., the median (Q2). Based on this patch size analysis, 21 × 21 is determined as the optimal patch size where the IQRs of the Bhattacharyya coefficient and Kullback–Leibler divergence decrease by less than 10% compared with the previous patch size (note that these two measures estimate precisely the similarities of the histograms).

Finally, considering from the hypothesis to the cost function in the previous section, a single patch can possess its own gradient distribution, and the corresponding cost function is derived in the same manner. A “small” cost function constructed by a single patch is represented as follows:

(15) $$f_{i}\left(x\right)={\left|\left|P_{i}\left(y-\mathrm{Hx}\right)\right|\right|}_{2}^{2}+\tilde{\lambda }{\left|\left|\nabla^{t}P_{i}\left(x\right)\right|\right|}_{2}\;,$$

where $f_{i}$ denotes a cost function considering the *i*-th patch, and $\tilde{\lambda }$ denotes the regularization parameter. The cost function in Equation (11) will be minimized by the successive minimization of these “small” cost functions in the next section.

### 3.3. Pixel-Wise Optimization

The hypotheses corresponding to each patch can be effectively employed by the adaptive iterative algorithm because the adaptive process considers the spatially varying local characteristics. to consider local priors, we have extended our previous work [22] to incorporate the hypotheses in the middle of minimizing the cost functions. In other words, we propose an optimization method based on the coordinate descent method, which is closely related to the adaptive process. The proposed method focuses on the coordinate-wise (or pixel-wise) image restoration in which a single pixel is updated by its own subproblem. The cost function of Equation (11) will be addressed to derive the subproblems of minimizing Equation (15), and each pixel is restored by solving the corresponding subproblem.

Typically, iterative algorithms are attractive for image restoration because they allow for the incorporation of prior knowledge and are fairly robust with respect to errors in the approximation of image degradations [31]. The proposed regularized optimization for the LWIR images departs from minimizing the cost function of Equation (11). The steady-state solution of our optimization problem is estimated by an iterative algorithm. Our update strategy is based on the coordinate-wise optimization and is represented as follows:

(16) $$x^{\left(k+1\right)}=x^{\left(k\right)}+\varepsilon \cdot e_{k\;\mathrm{mod}\;N},$$

where *N* is the number of LWIR images, $e_{i}$ denotes the standard basis, whose *i*-th component is one and the others are zero, mod denotes the modulo operation, and ε is an additive scalar to be added to the (kmodN)-th pixel.

The update strategy of Equation (16), in which a single pixel is updated in a single iteration, can be regarded as a projection onto a hyperplane [32]. The projection onto the hyperplane containing adjacent pixels allows the multivariable problem to be converted to a series of single-variable problems. The coordinate-wise optimization for updating each pixel in the LWIR image is derived as follows:

(17) $$\begin{array}{cc} \min F(x^{\left(k+1\right)}) & =\min_{\varepsilon }F\left(x^{\left(k\right)}+\varepsilon \cdot e_{k\;\mathrm{mod}\;N}\right)\\ \; & =\min_{\varepsilon }f_{\left(k\;\mathrm{mod}\;N\right)}\left(\varepsilon \right), \end{array}$$

where *F* denotes the multivariable cost function of Equation (11) and *f* denotes a simplified cost function of Equation (15) based on which the (kmodN)-th pixel is restored. This coordinate-wise and iterative algorithm progresses based on a sequence of scalar optimizations, i.e., at each iteration, a subproblem is derived and solved to calculate the additive scalar.

A subproblem of the isotropic TV-regularized cost function can be derived from Equation (15) with t=2 based on its projection onto a certain hyperplane. The derivation of the isotropic TV-regularized subproblems and the general form of the subproblems can be represented as below:

(18) $$\begin{array}{cc} F(x^{\left(k\right)}+\varepsilon \cdot e_{\tau })\; & ={\left\|y-H(x^{\left(k\right)}+\varepsilon \cdot e_{\tau })\right\|}_{2}^{2}\\ \; & \;+\tilde{\lambda }{\left\|\nabla^{t}\left(x^{\left(k\right)}+\varepsilon \cdot e_{\tau }\right)\right\|}_{2}\\ \; & =D_{2}\cdot \varepsilon^{2}-2D_{1}\cdot \varepsilon +D_{0}\\ \; & \;+\tilde{\lambda }\sqrt{R_{2}\cdot \varepsilon^{2}-2R_{1}\cdot \varepsilon +R_{0}}\\ \; & =d\left(\varepsilon \right)+\tilde{\lambda }\cdot r\left(\varepsilon \right)\\ \; & =f_{\tau }\left(\varepsilon \right), \end{array}$$

where τ is a substitution of (kmodN) for the coordinates calculated from the number of pixel updates. The subproblems can be divided into two parts: the quadratic equation from the data fidelity term, d(ε), and the irrational equation from the isotropic TV regularization term, r(ε). The coefficients $D_{m}$s of the data fidelity function are obtained by considering the neighborhood of the updated pixel as follows:

(19) $$\begin{array}{cc} D_{1} & =\sum_{u,v=-l}^{l}h_{\left(u,v\right)}\left(y_{\left(u,v\right)}-\sum_{u,v=-l}^{l}h_{\left(u,v\right)}x_{\left(i-u,j-v\right)}^{k}\right),\\ D_{2} & =\sum_{u,v=-l}^{l}h_{\left(u,v\right)}^{2}, \end{array}$$

where (i,j) denotes the coordinate of the updated pixel, and the $R_{m}$s of the regularization function with t=1 are obtained as follows:

(20) $$\begin{array}{cc} R_{0} & =\sum_{m,n=-l}^{l}{\left(\nabla x\right)}_{\left(i+m,j+n\right)}^{2},\\ R_{1} & =3x_{\left(i,j\right)}-x_{\left(i-1,j\right)}-x_{\left(i+1,j\right)}-x_{\left(i,j-1\right)}\\ \; & \;-x_{\left(i,j+1\right)}+\frac{1}{2}x_{\left(i,j\right)}+\frac{1}{2}x_{\left(i,j\right)},\\ R_{2} & =1.5, \end{array}$$

where ${\left(\nabla x\right)}_{\left(i,j\right)}$ denotes the (i,j)-th component of $\nabla x^{\left(k\right)}$. In the case of the second derivative, the coefficients of the regularization function are as below:

(21) $$\begin{array}{cc} R_{0} & =\sum_{m,n=-l}^{l}{\left(\nabla^{2}x\right)}_{\left(i+m,j+n\right)}^{2},\\ R_{1} & =\frac{3}{2}\left(x_{\left(i-1,j\right)}+x_{\left(i+1,j\right)}+x_{\left(i,j-1\right)}+x_{\left(i,j+1\right)}\right)\\ \; & -(x_{\left(i-1,j-1\right)}+x_{\left(i-1,j+1\right)}+x_{\left(i+1,j+1\right)}+x_{\left(i+1,j-1\right)})\\ \; & -\frac{1}{2}\left(x_{\left(i-2,j\right)}+x_{\left(i+2,j\right)}+x_{\left(i,j+2\right)}+x_{\left(i,j-2\right)}\right),\\ R_{2} & =1.25. \end{array}$$

**Figure 7:**
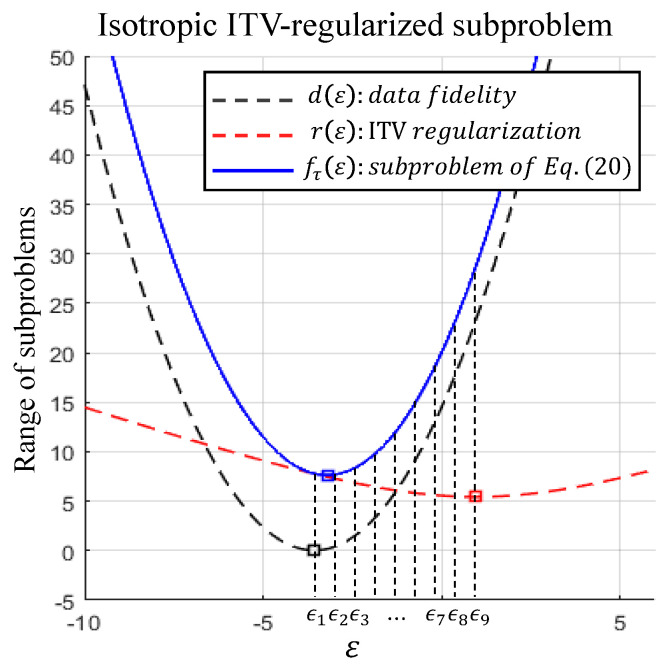
Example of a one-dimensional subproblem represented in Equation (18). A single variable subproblem (blue line) derived from the TV-regularized cost function of Equation (11) can be decomposed into two parts: the part derived from the data fidelity term (black line), and from the regularization term (red line). The solution of the subproblem lies between the critical points of data fidelity and the regularization functions. Nine points marked by $\epsilon_{l},\;\left\{l|1,2,\cdots ,9\right\}$ are investigated to find the minimum value of $f_{\tau }\left(\varepsilon \right)$. All functions are plotted on the same scale ($\tilde{\lambda }=1$) for visualization.

As in our previous work [22], the cost function of Equation (15) can be minimized by solving a series of subproblems demonstrated in Equation (18). The subproblem derived from the proposed regularized cost function can also be regarded as a one-dimensional regularized function, and the simplified version of a single variable function can be solved easily. Each pixel possesses its own subproblem, and the subproblems are solved by considering the algebraic characteristics. The version of the isotropic TV-regularized subproblem is described in Figure 7. We have observed that the final solution (blue rectangle) of the subproblem minimization is always located between the critical point of the data fidelity function (black rectangle) and of the regularization function (red rectangle). This characteristic can be verified using the mean value theorem. to minimize the function of Equation (18), several points between the vertices of the data fidelity and the regularization function are investigated; nine points placed at equal intervals in the range of $\left[\frac{D_{1}}{D_{2}},\frac{R_{1}}{R_{2}}\right]$ in our implementation. The final solution is substituted with the point of the minimum function value. The sequence of the pixel-wise optimizations can be varied with several sampling strategies, and the sequences are randomly constructed based on permutations as in [22]. Finally, our pixel-wise update strategy is represented as below:

(22) $$x^{\left(k+1\right)}=x^{\left(k\right)}+\varepsilon \cdot e_{\sigma (k\;\mathrm{mod}\;N)},$$

where σ(·) denotes a random permutation of size *N*, and ε minimizes the function of Equation (18). The proposed optimization strategy is demonstrated in Algorithm 1.
**Algorithm 1** Minimization of Equation (11)**Input:** Observed LWIR image y; iteratively estimated LWIR images $x^{\left(k\right)}$; system matrix H; regularization parameter λ as a constant; image size *N*; random permutations σ of size *N*; Threshold Th(=0.07 * MAX Intensity).**Output:** Estimated intrinsic LWIR image $\hat{x}$
  *Initialization*: $x^{\left(0\right)}=y$
  *LOOP Process*
1:**for**k=0 to *T* **do**2: **if** kmodN==0 **then**3:  σ(·)←generatePermutation(·)4: **end if**5: $\varepsilon \leftarrow pixelwiseOpt(y,H,x^{\left(k\right)},\sigma \left(k\;\mathrm{mod}\;N\right))$6: $x^{\left(k+1\right)}\leftarrow x^{\left(k\right)}+\varepsilon \cdot e_{\sigma (k\;\mathrm{mod}\;N)}$7: **if** $F\left(x^{\left(k\right)}\right)-F\left(x^{\left(k-N\right)}\right)<Th$ **then**8:  break;9: **end if**10:**end for**11:**return** $\hat{x}=x^{\left(k\right)}$

## 4. Results and Discussion

Several experiments have been conducted to quantify the effectiveness of our hypothesis and the optimization method. In general, the linear image-observation model of Equation (1) attempts to explain various degradation problems. In this study, the proposed optimization method is applied to denoising (H=I) and deconvolution, and thermal images are restored by incorporating natural statistics as the regularization strategies.

### 4.1. Measurements

**Figure 8:**
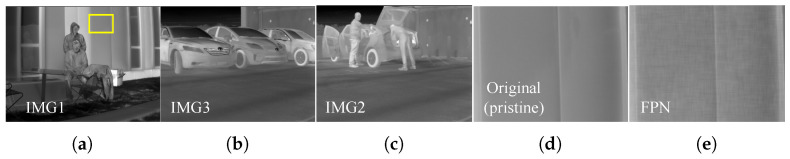
Images used for simulations (refer Table 4 and Table 5), and visualized noise. (**a**–**c**) original pristine images; (**d**) magnified region of image 1 (pristine image); (**e**) RNU of ($B_{u}$, $B_{v}$, $\sigma_{u}$, $\sigma_{v}$) = (20, 20, 25, 25).

The experiments reported herein, have tried to demonstrate restoration results from real-world LWIR images. In addition, some experiments have demonstrated the results of simulations quantifying the performance of LWIR restoration algorithms with the use of synthesized LWIR images. The pristine LWIR images have been contaminated with certain distortion as in [15], and the pristine images and noise for the simulations are represented in Figure 8. The pristine LWIR images are synthesized with RNU, and restored by the conventional and proposed algorithms. The results of these simulations are clearly demonstrated by image quality assessments, such as the peak signal-to-noise ratio (PSNR), and structural similarity index measure (SSIM) [33]. Moreover, we have utilized image quality assessments, the Roughness (Ro) [34] index and the Effective Roughness (ERo) [35] index, for real-world LWIR images that have no reference images to be compared to. In general, the no-reference assessments, Ro and ERo, are frequently used to quantify the performance of thermal image processing algorithms.

To quantify the performance of restoration algorithms for thermal images, the RNU proposed in [36] has been employed to our experiments. The RNU are frequently observed as grid-like patterns and differ from the additive white Gaussian noise (AWGN). The noise model that considers the thermal image can be represented in the frequency domain as below:

(23) $$\begin{array}{cc} |\tilde{I}\left(u,v\right)|= & B_{u}exp\left(\frac{-{\left(u-u_{0}\right)}^{2}}{2\sigma_{u}^{2}}\right)+\\ \; & \;\;\;\;B_{v}exp\left(\frac{-{\left(v-v_{0}\right)}^{2}}{2\sigma_{v}^{2}}\right),\\ ∠\tilde{I}\left(u,v\right) & ∼U[-\pi ,\pi ], \end{array}$$

where $u_{0}$, $B_{u}$, and $\sigma_{u}$ (correspondingly, $v_{0}$, $B_{v}$, and $\sigma_{v}$) are the location, the amplitude, and the scale of the horizontal band (correspondingly, vertical), and *U* denotes the uniform distribution. In our experiments, the severity of RNU has been controlled by manipulating $B_{u}$, $B_{v}$, $\sigma_{u}$, and $\sigma_{v}$.

### 4.2. Regularization Strategy for Thermal Images

The proposed natural statistics of the thermal image are clarified using Equations (10) and (14). The regularization strategy for images obeying the Laplace distribution is suggested as the anisotropic TV, and thermal images is verified to obey the Laplace distribution in our experiments. Moreover, the properties of Equations (7) and (8) yield the isotropic TV rather than the anisotropic TV for the various LWIR datasets. In our previous work [22], the cost function regularized by the anisotropic TV was minimized by the general solution for each pixel-wise optimization process, but the proposed method minimizes the isotropic TV-regularized cost function by sweeping the certain range (refer to Figure 7). To explicitly propose a proper regularizer for thermal images, we have conducted an experiment based on the use of the pristine images as the reference images. Furthermore, the pristine images and the condition of noise in Figure 8 have been employed. The synthesized images have been restored under three conditions: RNU of ($B_{u}$, $B_{v}$, $\sigma_{u}$, $\sigma_{v}$) = (20, 20, 25, 25).

To demonstrate the natural statistics clearly, we have established several regularized cost functions. Each cost function is characterized by its regularization function. The regularization strategies for the LWIR image can be derived from both sides of Equation (10). The first regularization function, which is often called anisotropic TV, is expressed as below:

(24) $$\Gamma_{aniso}\left(x\right)=\left|\nabla x\right|,$$

and the second regularization function, which is often called isotropic TV, is as below:

(25) $$\Gamma_{iso}\left(x\right)={\left||\nabla x|\right|}_{2}.$$

The regularization strategies with TV [26,28] are often used for noise reduction. Additionally, two regularization functions, derived from both sides of Equation (10), are added to our list by employing the second derivatives. The second derivative is often called the Laplacian operator, and the regularization function with the L1-norm is marked with “*L1Lap*”, and as below:

(26) $$\Gamma_{L1Lap}\left(x\right)=\left|\nabla^{2}x\right|.$$

Correspondingly, the regularization function of the Laplacian operator with the L2-norm is marked with “*L2Lap*”, and is as below:

(27) $$\Gamma_{L2Lap}\left(x\right)=\left|\right|\nabla^{2}x{\left|\right|}_{2}.$$

Note that in the case of the thermal image, the regularization functions of Equations (24) and (26) can be substituted with Equations (25) and (27), respectively.

**Figure 9:**
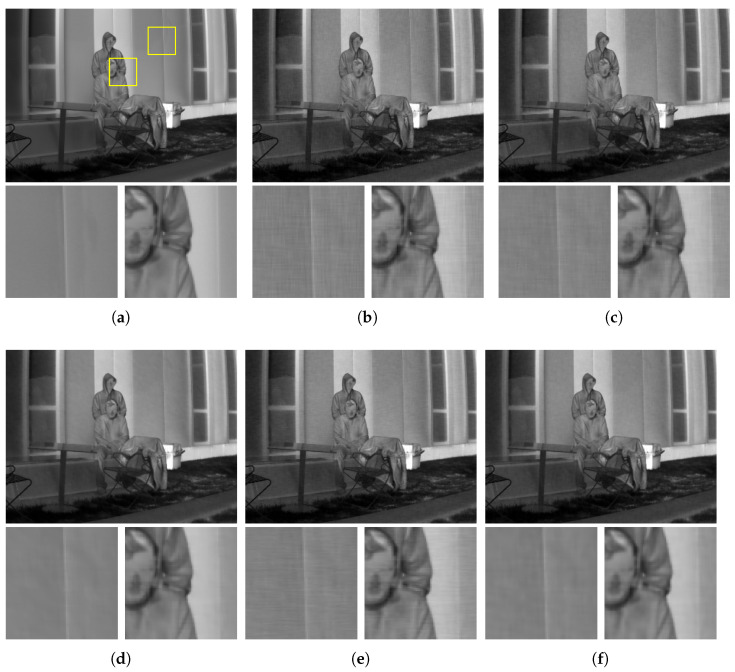
Results from the experiment of RNU. Regions marked with rectangles are magnified. The cost functions incorporating our hypothesis demonstrate more clear output images. (**a**) Original image; (**b**) degraded image; (**c**) result with Equation (24); (**d**) result with Equation (25); (**e**) result with Equation (26); (**f**) result with Equation (27).

To propose an optimal regularization strategy for thermal images, LWIR images have been restored with the four regularizers. The suggested regularization strategies can be categorized into two groups: regularization using the $L_{1}$-norm (Equations (24) and (26)) and $L_{2}$-norm (Equations (25) and (27)). Table 4 demonstrates the noise immunity of the four regularization strategies. Figure 9 visualizes the results of the four regularization strategies. The second group that incorporating suggested thermal image statistics outperforms the others both qualitatively and quantitatively. The results of the first group still demonstrate remaining noise after restoration. However, the second group reduces RNU effectively. Especially, isotropic TV outperforms the others in both flat and edge regions. Therefore, Equation (25) is proposed as an optimal regularizer for noise reduction in thermal images.

### 4.3. Final Results and Discussion: Denoising

In the final results, as mentioned above, the proposed method reduces noise using the optimal regularization strategy, i.e., isotropic TV. Several conventional methods have been compared with the proposed method, and both the synthesized images and real-world images have been restored to quantitatively demonstrate the results. We attempted to find algorithms that had been implemented for thermal images, but conventional methods for thermal imaging seldom exist. Typical degradation problems of thermal images are explained as RNU (or FPN). The condition of RNU is different from AWGN and device-dependent, but most of denoising algorithms are implemented for removing AWGN from the visible light images. Therefore, we tried to appropriately quantify our method by comparing with the widely known conventional methods to objectively demonstrate the results.

The list of the conventional algorithms consists of BM3D [37], split Bregman method [38], Ochs et al. [39], and TWSC [40]. BM3D [37] represents the methods based on block matching in which image patches are grouped together based on similarity. The split Bregman method, representing the TV-regularized optimization methods, employs primal and dual variables, and projection onto the feasible set. Ochs et al. represents optimization method incorporating non-smooth and non-convex cost functions. TWSC is a method based on sparse coding. The parameters in their algorithms were suitably set to generate the best possible results. The grid-like patterns of RNU were sometimes recognized as not the noise but a part of high-frequency texture, and the patterns were enhanced by the restoration process. In this case, the results were selected from over-smoothed or pattern-enhanced results based on the scores of the measurements.

**Figure 10:**
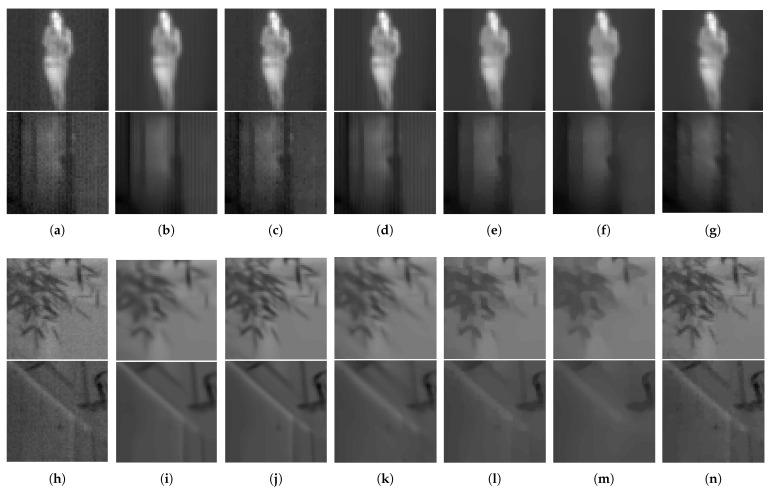
Final results of the conventional and proposed method. (**a**,**h**) Original degraded image of MORRIS (**top**) and KRISTO dataset (**bottom**); (**b**,**i**) BM3D; (**c**,**j**) Ochs et al.; (**d**,**k**) TWSC; (**e**,**l**) split Bregman with ATV; (**f**,**m**) split Bregman with ITV; (**g**,**n**) proposed method.

To quantify the final results, two experiments have been conducted: first with the synthesized images and reference image assessments, and second with the real-world images and no-reference image assessment. Table 5 demonstrates the results of the first experiment. In the case of RNU, which is inherent degradation of thermal imaging, ITV-based method outperforms the others. Real-world thermal images are restored in the second experiment. Figure 10 shows the restored images in detail. In the case of the severe RNU, the grid-like patterns have been recognized as detailed textures by some conventional methods, BM3D, Ochs et al., TWSC. However, the TV-based methods, including the proposed method, effectively remove severe RNU patterns. In this experiment, we can observe the difference of the results from both anisotropic and isotropic TV. The results of TWSC also shows the effective regularizer. The isotropic TV reduces noise in thermal image more effectively than anisotropic TV in both flat and edge regions. This tendency can be observed in Table 6 demonstrating the quantitative analysis of the noise removal algorithms. The ITV-based methods have recorded lower scores of Ro and ERo, and the proposed method shows lower scores in many datasets (note that Ro and ERo could not be regarded as imperative assessments, but they have been widely used to measure the quality of thermal images).

### 4.4. Application: Deconvolution

**Figure 11:**
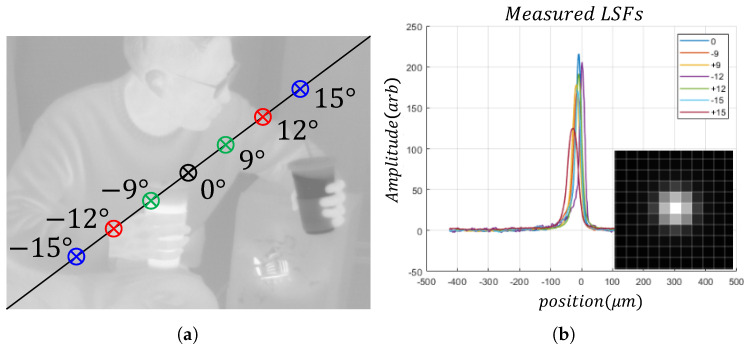
Application to LWIR image deconvolution. (**a**) Points of measured LSFs. The measured LSFs were obtained by varying the distance from the optical axis. Seven LSFs were measured at the points marked with ⊗ by the optical device; (**b**) visualized LSFs and their PSF. The PSF is reconstructed by the Radon transform [41] based on the LSFs. Averaging of LSFs and A2D conversion considering the pixel size of the LWIR sensor have been utilized to reconstruct the PSF.

**Figure 12:**
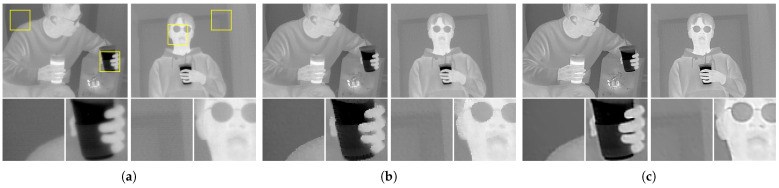
Results of image deconvolution. Regions marked with rectangles are magnified. In the case of image deconvolution, isotropic TV has generated clear output images. (**a**) Degraded original image; (**b**) restored based on the regularization term of Equation (24); (**c**) restored based on the regularization term of Equation (25).

The natural statistics of the thermal images can also be employed for image deconvolution. In this case, the image observation model describes the degradation of linear blurring. For example, defocus aberration or atmospheric turbulence blur can be included in this problem. In this study, the SRIP dataset, which consists of real-world images, is restored based on the observed point spread function (PSF). To demonstrate degradation, line spread functions (LSFs) were obtained at each region within the field of view as demonstrated in Figure 11. Note that the PSF has been constructed based on the LSFs, and our PSF indicates the optical characteristics of our device. As in the denoising results, isotropic TV regularization can be regarded as a proper strategy for thermal image deconvolution. Figure 12 shows the thermal images restored by the two TV regularization strategies. Isotropic TV regularization generates clearer output images, and anisotropic TV regularization generates over-smoothed results.

## 5. Conclusions

LWIR sensors have their own optical and electrical characteristics, and some statistical properties have been studied for a decade. However, despite these studies, the characteristics of thermal images have seldom been incorporated into thermal image processing. In this study, a statistical property of thermal imaging sensors has been derived, and the generality of the property is verified with various datasets. Moreover, the cost function considering the natural statistics is also proposed. The gradient statistics explains thermogram from various LWIR sensors without considering the individual sensor characteristics. The regularization strategies consider the natural statistics, and the specialized pixel-wise optimization restores thermal images effectively. During the COVID-19 pandemic, we hope that the proposed method will contribute to the generation of high-quality thermal images.

## Figures and Tables

**Table 1:** Definition of Measures qualifying the similarity between histograms of the gradient distribution.

	Definition	Range	Remarks
Correlation coefficient (ρ)	$\rho_{h_{p},h_{q}}=\frac{E[\left(h_{p}-\mu_{h_{p}}\right)\left(h_{q}-\mu_{h_{q}}\right)]}{\sigma_{h_{p}}\sigma_{h_{q}}}$	[−1,1]	$\rho_{h_{p},h_{p}}=1$
Histogram intersection (∩)	$h_{p}\cap h_{q}=\sum_{i}\min\left(h_{p}\left(n\right),h_{q}\left(n\right)\right)$	[0,1]	$h_{p}\cap h_{p}=1$
Chi-squared test $\left(\chi^{2}\right)$	$\chi^{2}\left(h_{p},h_{q}\right)=\frac{1}{2}\sum \frac{{\left[h_{p}\left(n\right)-h_{q}\left(n\right)\right]}^{2}}{\left[h_{p}\left(n\right)+h_{q}\left(n\right)\right]}$	[0,1]	$\chi^{2}\left(h_{p},h_{p}\right)=0$
Bhattacharyya distance $\left(D_{B}\right)$	$D_{B}\left(h_{p},h_{q}\right)=-\ln\left(\sum_{i}\sqrt{h_{p}\left(n\right)h_{q}\left(n\right))}\right)$	[0,*∞*)	$D_{B}\left(h_{p},h_{p}\right)=0$
Kullback–Leibler divergence $\left(D_{KL}\right)$	$D_{KL}(h_{p}\left|\right|h_{q})=\sum_{i}h_{p}\left(n\right)\ln\left(\frac{h_{p}\left(n\right)}{h_{q}\left(n\right)}\right)$	[0,*∞*)	$D_{KL}(h_{p}\left|\right|h_{p})=0$

**Table 2:** Average scores of the measures in Figure 2. The scores of the correlation coefficient and histogram intersection are close to unity, and the scores of the Chi-squared test, Bhattacharyya distance, and Kullback–Leibler divergence are close to zero.

	MOR	KRI	ADAS	SRIP	OSU	Average
ρ	0.9867	0.9722	0.9814	0.9808	0.9551	0.9757
∩	0.9055	0.8764	0.9074	0.8758	0.8768	0.8897
$\chi^{2}$	0.0249	0.0287	0.0149	0.0325	0.0285	0.0256
$D_{B}$	0.0151	0.0164	0.0081	0.0195	0.0157	0.0148
$D_{KL}$	0.1498	0.1312	0.0462	0.1605	0.0780	0.1132

**Table 3:** Averaged scores from three filtered images in Figure 2. Similar to the gradients, the scores of the correlation coefficient and histogram intersection are close to unity, and the scores of the Chi-squared test, Bhattacharyya distance, and Kullback–Leibler divergence are close to zero.

	MSCN	1st Derivative	2nd Derivative
ρ	0.9778	0.9754	0.9701
∩	0.8587	0.8954	0.8444
$\chi^{2}$	0.0424	0.0258	0.0524
$D_{B}$	0.0252	0.0150	0.0316
$D_{KL}$	0.1250	0.1235	0.1376

**Table 4:** Thermal image denoising using synthesized images with the four regularization strategies. Performances of each regularizer have been measured by four image assessments: PSNR and SSIM.

	PSNR(dB)			SSIM		
	IMG1	IMG2	IMG3	IMG1	IMG2	IMG3
degraded	39.14	39.15	39.13	0.9315	0.9255	0.9267
Equation (24)	39.86	40.15	40.13	0.9440	0.9441	0.9469
Equation (25)	40.57	41.59	41.74	0.9565	0.9646	0.9706
Equation (26)	39.67	40.03	40.11	0.9413	0.9424	0.9454
Equation (27)	40.40	41.13	41.16	0.9548	0.9607	0.9655

**Table 5:** Thermal image denoising using synthesized images with the conventional algorithms. Performances of each algorithm have been measured by four image assessments: PSNR, SSIM, Ro, and ERo.

	PSNR			SSIM			Ro			ERo		
	IMG1	IMG2	IMG3	IMG1	IMG2	IMG3	IMG1	IMG2	IMG3	IMG1	IMG2	IMG3
Degraded	39.14	39.15	39.13	0.9315	0.9255	0.9267	0.1019	0.0610	0.0789	0.4866	0.2275	0.2348
BM3D	39.14	39.15	39.13	0.9333	0.9290	0.9316	0.0928	0.0561	0.0738	0.4427	0.2075	0.2182
Ochs	40.17	40.38	40.41	0.9491	0.9477	0.9503	0.0791	0.0465	0.0623	0.3833	0.1736	0.1856
TWSC	39.56	39.4	39.60	0.9370	0.9293	0.9350	0.0787	0.0538	0.0701	0.3777	0.1996	0.2079
SB(ATV)	40.63	41.24	41.77	0.9550	0.9641	0.9612	0.0626	0.0342	0.0470	0.2989	0.0920	0.1329
SB(ITV)	40.85	41.23	41.27	0.9588	0.9607	0.9533	0.0384	0.0372	0.0524	0.3063	0.1384	0.1565
Equation (25)	40.57	41.59	41.74	0.9565	0.9646	0.9706	0.0490	0.0301	0.0445	0.2416	0.1105	0.1325
Equation (27)	40.40	41.13	41.16	0.9548	0.9607	0.9655	0.0553	0.0338	0.0485	0.2681	0.1241	0.1439

**Table 6:** Thermal image denoising using real-world images with the conventional algorithms. Performances of each algorithm have been measured by no-reference image assessments.

	Ro					ERo				
	MORR.	KRIS.	ADAS	SRIP	OUS	MORR.	KRIS.	ADAS	SRIP	OUS
Degraded	0.0418	0.0861	0.1455	0.2539	0.0456	1.2411	0.3905	0.5043	0.4351	0.2670
BM3D	0.0314	0.0640	0.1432	0.2522	0.0376	0.8530	0.2907	0.4961	0.4324	0.2148
Ochs	0.0220	0.0568	0.1313	0.2475	0.0332	0.6608	0.2662	0.4561	0.4266	0.1923
TWSC	0.0194	0.0505	0.0399	0.0308	0.2567	0.2886	0.2376	0.1454	0.1771	0.4243
SB(ATV)	0.0100	0.0353	0.0546	0.2168	0.0260	0.1877	0.1709	0.1946	0.3851	0.1473
SB(ITV)	0.0097	0.0314	0.0421	0.2097	0.0257	0.1722	1521	0.1502	0.3745	0.1403
Equation (25)	0.0093	0.0338	0.0495	0.2182	0.0251	0.1830	0.1413	0.1314	0.3869	0.1314
Equation (27)	0.0113	0.0422	0.0550	0.2327	0.0282	0.2266	0.1752	0.1895	0.3996	0.1526

## Data Availability

Datasets available online: https://vcg.seas.harvard.edu/publications/statistics-of-infrared-images and https://www.flir.com/oem/adas/adas-dataset-form/ (accessed on 14 July 2021).
